# Detecting ADHD through natural language processing and stylometric analysis of adolescent narratives

**DOI:** 10.3389/frcha.2025.1519753

**Published:** 2025-05-09

**Authors:** Juan Barrios, Elena Poznyak, Jessica Lee Samson, Halima Rafi, Simon Gabay, Florian Cafiero, Martin Debbané

**Affiliations:** ^1^ Developmental Clinical Psychology Research Unit, Faculty of Psychology and Educational Sciences, University of Geneva, Geneva, Switzerland; ^2^ Faculty of Humanities, University of Geneva, Geneva, Switzerland; ^3^ École Nationale des Chartes, PSL, Paris, France; ^4^ Research Department of Clinical, Educational and Health Psychology, University College London, London, United Kingdom

**Keywords:** ADHD, self-defining memories, emotion regulation, natural language processing, stylometry, computational linguistics

## Abstract

**Introduction:**

Attention-Deficit/Hyperactivity Disorder (ADHD) significantly affects adolescents' everyday lives, particularly in emotion regulation and interpersonal relationships. Despite its high prevalence, ADHD remains underdiagnosed, highlighting the need for improved diagnostic tools. This study explores, for the first time, the potential of Natural Language Processing (NLP) and stylometry to identify linguistic markers within Self-Defining Memories (SDMs) of adolescents with ADHD and to evaluate their utility in detecting the disorder. A further novel aspect of this research is the use of SDMs as a linguistic dataset, which reveals meaningful patterns while engaging psychological processes related to identity and memory.

**Method:**

Our objectives were to: (1) characterize linguistic features of SDMs in ADHD and control groups; (2) assess the predictive power of stylometry in classifying participants' narratives as belonging to either the ADHD or control group; and (3) conduct a qualitative analysis of key linguistic markers of each group. Sixty-six adolescents (25 diagnosed with ADHD and 41 typically developing peers) recounted SDMs in a semi-structured format; these narratives were transcribed for analysis. Stylometric features were extracted and used to train a Support Vector Machine (SVM) classifier to distinguish between narratives from the ADHD and control groups. Linguistic metrics such as wordcount, lexical diversity, lexical density, and cohesion were computed and analyzed. A qualitative analysis was also applied to examine stylistic patterns in the narratives.

**Results:**

Adolescents with ADHD produced narratives that were shorter, less lexically diverse, and less cohesive. Stylometric analysis using an SVM classifier distinguished between ADHD and control groups with up to 100% precision. Distinct linguistic markers were identified, potentially reflecting difficulties in emotion regulation.

**Discussion:**

These findings suggest that NLP and stylometry can enhance ADHD diagnostics by providing objective linguistic markers, thereby improving both its understanding and diagnostic procedures. Further research is needed to validate these methods in larger and more diverse populations.

## Introduction

ADHD is a neurodevelopmental disorder that affects between 5.6% and 8% of youth between 12 to 18 years (1, 2). Its causes are multifactorial: several genetic and environmental risk factors act together to increase susceptibility to this disorder and other psychiatric comorbidities (3). During adolescence, individuals with ADHD are particularly prone to challenges in emotion regulation and in interpersonal relationships (4). A significant characteristic of these difficulties is their tendency to combine and mutually reinforce. For instance, difficulties within the social domain are often exacerbated and amplified by deficiencies in emotional regulation and pragmatic language abilities (5). These issues often result in low self-esteem, social problems, increased risks of substance abuse (6), peer rejection, social isolation, or academic failure (7).

Despite its high prevalence and impact, ADHD is still relatively underdiagnosed in most countries (8). Additionally, it is well known that early detection and treatment of ADHD are effective strategies for managing its course and mitigating long-term impacts (9). Focusing on detection during adolescence offers therefore an opportunity to facilitate earlier interventions, reduce risks, and improve outcomes. However diagnosing ADHD is a complex and time-consuming process requiring a comprehensive and multidisciplinary assessment (10). This involves evaluating clinical history, using standardized rating scales, gathering school information, and applying DSM-5 or ICD-10 criteria to assess symptoms across various contexts while excluding other mental disorders with overlapping symptoms (3). Along with that, the effectiveness of ADHD standardized rating scales can be compromised by informant biases (11), where different respondents (e.g., parents, teachers, or individuals themselves) may rate the same behaviors differently due to subjective viewpoints or contextual experiences [cf. (12)]. This variability can lead to inaccurate assessments of ADHD symptoms (11) and underscores the importance of using both subjective measures (e.g., self-reports and observer ratings) and objective measures (e.g., Continuous Performance Test) to improve diagnostic accuracy and reliability (13, 14).

With the rise of Artificial Intelligence (AI) in the last decade, other objective methods to gather information have emerged like computer-based linguistic methods from the field of Natural Language Processing (NLP). These approaches offer objective measures through the analysis of text and speech features, thereby mitigating the inherent subjectivity of traditional standardized rating scales (15), with reduced implications in terms of time, cost, and infrastructure required for its deployment. For more than ten years now, NLP has been used in neuroscience and psychiatry [cf. for example, (16–19)]. In fact, the latest research in this field makes it possible not only to identify mental health risks like suicidal risk behaviour (20) but can also contribute to predict the onset of mental disorders on a linguistic basis (21–23). Considering these facts, one can say that AI and NLP techniques have emerged as robust tools for various clinical applications, demonstrating their efficacy in diverse contexts. With their ability to analyze and interpret linguistic patterns and to capture and process language use in different contexts, AI and NLP offer a direct approach to gather clinical insights that hold promise in revolutionizing the screening and diagnostic processes for ADHD and other mental-health disorders.

Research at the intersection of NLP and psychology extends beyond English to analyze various languages such as Spanish (22, 24), Chinese (25), French (26), and Korean (27, 28). Two Korean language studies analyzed the language patterns of individuals with ADHD across different age groups and contexts. The first study (27) compared language use in children diagnosed with combined ADHD with a non-clinical control group. Results showed significant between-group differences in word use and language style of both groups thereby highlighting possible distinctive linguistic markers for combined ADHD in childhood. Building on these preliminary findings, the second study (28) extended the investigation to Korean college students with ADHD symptoms and revealed the persistence of a distinct language style associated with ADHD across different developmental stages.

In the context of the present study, these findings are fundamental: if a specific language style of ADHD can be detected, then stylometric methods have the potential to detect it in other languages too and, therefore, enhance the accuracy and efficiency of preliminary screenings and/or diagnoses of ADHD (29–32) and contribute to simplify its procedure. Indeed, stylometry has its own techniques specifically oriented to detect language styles and has already proven to be very efficient in literature (33), forensic science (34) or social media studies (35). Furthermore, it also gives researchers the opportunity to examine stylometric markers in relation to clinical conditions (36).

Self-defining memories (SDMs) represent a specific type of autobiographical memory associated with the self-concept (37, 38), contributing to an individual’s sense of coherence and of continuity (39). Consequently, SDMs are conceptualized as fundamental components of personal identity at cognitive, motivational, and affective levels. SDMs are particularly useful in the fields of stylometry and NLP for detecting mental health disorders because they often encapsulate significant emotional experiences, making them rich sources of data. By examining the language used in these narratives, researchers can gain insights into an individual’s cognitive and emotional processes (40). In stylometry, SDMs allow for the examination of linguistic patterns and their relationship with psychological distress or well-being. Furthermore, SDMs provide a consistent and structured format for collecting data across different individuals by means of the SDMs Task and thus enhancing the reliability and validity of the research findings.

This study has three primary objectives: our first aim is to employ NLP to compare the linguistic features of SDMs in adolescents with ADHD to typically developing peers. Our second goal is to quantify the predictive power of stylometry for group classification, distinguishing between controls and ADHD individuals. Finally, our third objective is to conduct a qualitative analysis of the key linguistic markers identified. The latter is crucial for several reasons. On the one hand, qualitative analysis offers a deeper understanding of the context and nuances of language use, which purely quantitative methods might overlook (41). On the other hand, qualitative insights can help validate and interpret the results of quantitative analyses, ensuring that identified markers are not only statistically significant but also meaningful and relevant in real-world settings (42). This can increase the potential for these markers to be used in diagnostic and screening processes.

Based on the findings of the Korean studies mentioned above (27, 28), which identified a specific narrative style associated with individuals prone to ADHD, we hypothesized that this distinct narrative style would be detectable in the self-defining narratives of adolescents. Specifically, we expected that advanced stylometric techniques would unveil narrative patterns (i.e., markers) in individuals’ SDMs with ADHD.

## Materials and methods

### Participants

25 Adolescents with ADHD (ADHD group, 12 females and 13 males) and 41 without ADHD (control group, 22 females and 19 males) were included in the study. All adolescents were between 12 and 17 years old and fluent in French. For both groups, the exclusion criteria included a history of psychotic disorders, diagnosed personality disorder, autism spectrum disorder, or neurological disorders. In the ADHD group, these criteria were assessed during the clinical intake through an anamnestic interview conducted with the parents. In the control group, exclusion criteria were explicitly screened prior to participation through a standardized pre-task questionnaire.

#### ADHD group

Adolescents with ADHD were recruited as part of a research project conducted at the University of Geneva advertised in local parent associations for children with ADHD and through collaborations established with local child psychiatrists. Diagnostic criteria were investigated by detailed anamnestic interviews and confirmed using the ADHD Child Evaluation interview (43). All diagnostic assessments were conducted by experienced clinical psychologists specialized in ADHD.

#### Control group

Typically developing (TD) controls were recruited from the general population in Geneva through advertisements and personal referrals and received compensation for their participation. Specific inclusion criteria for this group required the absence of intellectual impairments, as assessed by the Block Design and Vocabulary subtests of the Wechsler Intelligence Scale for Children [WISC-IV; (44)].

Of note, no significant differences were found between the ADHD and control groups on the WISC-IV Block Design subtest, suggesting comparable performance between groups on these core measures of visuospatial reasoning and verbal comprehension (ADHD: M=10.73, SD=2.74; Control: M=11.85, SD=2.43; W=406.5, p=0.141) or the Vocabulary subtest (ADHD: M=12.27, SD=2.49; Control: M=12.31, SD=3.03; W=515.5, p=0.957).

### Sampling

#### Pairwise matching

Pairwise matching was used to balance the ADHD and the control groups with respect to the means of participants’ sex and age in order to make them comparable and get a more fine-grained analysis of the differences between the two groups. The two samples were matched by cardinality method (45) to find the largest matched set (in this case by age and sex) with the additional constraint that the ratio between the number of adolescents in both groups had to be equal to 1. This method minimizes between-group differences based on age or sex, while selecting the best-fitting control cases for the ADHD group with minimal loss of ADHD cases.

#### Final samples

In both groups the final sample meeting the pairwise inclusion criteria consisted of 24 adolescents in both groups (cf. Table 1). The mean age in the ADHD group was 15.14 (σ = 1.83) and 15.21 (σ = 1.44) for the control group. A Student two samples *t*-test showed that the difference was statistically not significant (*t*(43.65) = −0.16, *p*-value > 0.05). As a result of this pairwise matching, the total number of SDMs per group was 72, derived from 24 participants, each contributing with 3 SDMs.

**Table 1:** ADHD Characteristics of participants after pairwise matching.

Variable	ADHD (mean/count)	ADHD (SD/%)
Primary symptoms		
Inatention	7.61	1.2
Hyperactivity	4.61	2.78
ACE diagnosis		
ADHD-inattentive	15	62.5%
ADHD-hyperactive	1	4.2%
ADHD-combined	8	33.3%
Comorbities		
Language and/or Learning disorders	4	16.7%
Anxiety	2	8.3%
Conduct disorder	1	4.2%
Sleep disorder	1	4.2%
Medication		
On medication	9	36%

### Data

#### Self-defining memories (SDM)

The SDMs investigated in this study were collected with the SDM Task^1^ (37). During this task, participants were asked to recall personal memories of events with specific attributes, which will be described next. Participants were asked to write three SDMs. They were told that SDMs refers to important life events that occurred at least one year ago and helped them to understand who they are. Other characteristics of SDMs were also given to the participants: SDMs are generally vividly represented and meaningful, they generate strong feelings (positive or negative) and are often recalled by individuals on a voluntary basis or spontaneously. While listening to the description, participants had a printed summary in front of them outlining the main points of the task. Then they were told to imagine a situation where they meet someone they are fond of during a walk to share several personal past events that powerfully convey how they have become who they are today. The participants were then given three sheets of paper and asked to write down one SDM on each sheet. For each event, participants were asked to write a one sentence summary and a sufficiently detailed description to help the imagined friend see and feel as they did in the past. Afterward, participants rated their feelings after recalling each memory using a 7-point scale from 0 (not at all) to 6 (extremely) for positive and negative affects. Finally, they estimated how much time had passed since each event in years and months, thus providing a self-reported measure of the time frame for each SDM.

#### Final corpus

The corpus is made up of a series of 144 SDMs, collected in two samples, one consisting of 24 adolescents with a diagnosis of ADHD and the other with 24 control participants, who all had to write a total of three SDMs each. All texts were handwritten in French, and then transcribed by the person in charge of the experiment. Spelling mistakes were corrected at the time of entry, allowing the machine to focus on the deep structure of the language, and not on the vagaries of its form.

### Linguistic metrics

Among the various metrics used in NLP to analyze and quantify aspects of text in a psychological perspective, we chose word count, lexical density, lexical diversity and cohesion [cf. (40, 46–48)].

#### Wordcount

Is a quantitative measure of the number of tokens (: a computational approximation of the linguistic concept of “word,” defined as a string of characters between two delimiters, such as punctuation or spaces) present in a given text or set of texts. Depending on the definition of a token, this means that we count the number of words, punctuation marks, and/or symbols in a text (49).

In the context of this study, we operationalize wordcount using the definition of the token of the UDPipe package (50). The delimiters are spaces, tabulations, newlines, punctuation signs, apostrophes (*J’ai* = 2 tokens) and hyphens (*beau-père* = 2 tokens). The resulting wordcount is the total count of the number of units obtained after this tokenization process.

(1) $$\mathrm{Wordcount}=\sum_{i=x}^{N}x$$

Where:

*N* = Total number of tokens in the text

*x* = 1 token.

#### Lexical diversity

Refers to the variety of unique lexical items (types) used in a text (51) and serves as a metric for assessing the language proficiency of individuals. In psychological research, lexical diversity has been studied across various settings. For example, (52) explored Lexical Diversity among three groups of preschool-aged children (aged 3 to 5 years): (1) children with Specific Language Impairment (SLI) (2) age-matched children with Typical Development (TD) and (3) language-matched children with typical development (LM). Watkins et al. (52) findings consistently distinguished children with SLI from their TD and LM peers. Similarly, (53) investigated lexical diversity in children older than those of Watkins study (aged 5 to 8 years) in three groups: (1) children with ADHD, (2) children with SLI, and (3) typically developing children (TD). The study found that children with SLI demonstrated lower lexical diversity compared to both the ADHD and TD groups. Conversely, no significant difference in lexical diversity was found between the ADHD and TD groups.

In this study, we use the Moving-Average Type-Token Ratio (MATTR) method to assess the lexical diversity of the two groups. The Type-Token Ratio (TTR) is a traditional measure of lexical diversity, calculated by dividing the number of unique lexical items (types) by the total number of “words” (tokens) in a given text. The Moving-Average Type-Token Ratio (MATTR) refines this measure by calculating the TTR over a sliding window of fixed size, in this case 50 tokens, applied across the entire text. This window size is frequently used because it offers an optimal balance between measurement sensitivity and statistical reliability. The average of these windowed TTRs was then computed, providing a more reliable estimate of lexical diversity that accounted for variations in text length (54). This method improves accuracy and reliability by incorporating more data points than traditional methods for computing lexical diversity.

(2) $$\text{MATTR}=\frac{1}{N-W+1}\sum_{i=1}^{N-W+1}\frac{V_{i}}{W}$$

Where:

*N* = Total number of tokens in the text

*W* = Window size (number of tokens in each segment)

*Vi* = Number of unique tokens in the *i*-th segment.

We take the same mathematical definition of the lexical diversity [cf. Equation 2] that (52, 53).

#### Lexical density

Reflects the proportion of content words (nouns, verbs, adjectives, and adverbs) to the total number of tokens in a text (55). As such, it provide a measure of the amount of information in a given text (56). Lexical density evolves during human lifespan and it is influenced by different factors like emotional state (57) and the educational background (58). To calculate this network metric, content words are extracted from the corpus by filtering according to their part-of-speech annotations, which are generated using the udpipe R package (59).

In this study, we compute the Average Lexical Density (ALD) by segmenting the text, then we compute the ALD for each segment (55), and then we average all the results for each group (cf. Equation 3), ADHD and control.

(3) $$\text{ALD}=\frac{1}{n}\sum_{i=1}^{n}\left(\frac{\text{n( CW) in }S_{i}}{\text{n( TW) in }S_{i}}\times 100\right)$$

Where:

*CW* = Content words

*S* = Segment

*TW* = Total number of words in the i-th segment.

#### Cohesion

Analysis in NLP examines how well connected different parts of a text are connected (i.e., the use of pronouns, conjunctions, and lexical repetition), which help to maintain continuity and facilitates communication (60). High cohesion in narratives is often associated with better mental health and cognitive functioning. For example, individuals who use cohesive devices effectively tend to exhibit more organized thought processes and better narrative skills, which are indicative of a well-integrated self-concept and adaptive functioning (40). Conversely, low cohesion can signal disorganized thinking, which is often seen in various psychological disorders such as schizophrenia or severe anxiety disorder, where individuals may struggle to maintain a clear and connected narrative (61, 62). Along with this, lower cohesion is often observed in individuals with ADHD, reflecting their attentional challenges and cognitive variability.

Textual cohesion is calculated for each participant by analyzing specific cohesive devices—namely, parts of speech that are known to enhance cohesion—pronouns, adverbs, determiners, and coordinating conjunctions [cf. (63)]. Cohesive devices are counted at the participant level (3 SDMs), not the text level (1 SDM), to obtain a Cohesion Score (CS) per person (cf. Equation 4).

(4) $$CS_{\text{\textbackslash{},participant}}=\frac{1}{n_{i}}\sum_{\;j=1}^{n_{i}}T_{i,j}$$

Where:

$n_{i}$ = Number of cohesive devices in document i

$T_{i,j}$ = Total number of cohesive devices for participant i in part-of-speech category j.

Subsequently, the mean and standard deviation for each group are calculated based on the individual scores of the participants.

### Machine learning for textual analysis

We decided to use a classical machine learning method rather than deep learning, because according to a recent survey (64) the former performs better than the latter for profiling in similar settings (short texts, boolean or few categories). Because we were confident in ADHD diagnoses of participants, we decided to rely on a supervised method—otherwise, an unsupervised method would have been more appropriate (65). Among the supervised solutions available, we choose a standard algorithm in stylometry: Support Vector Machines (SVM) and not random forest (66) or logistic regression (67, 68), as SVM allows for easy interpretation and is established as a standard method in stylometry (69–72).

All participants’ SDMs are collected in a single file for each group, with the exception of those written by two people from the control group and two from the ADHD group for a final blind test.

The analyses were implemented with the SuperStyl package (73), which has been used in previous studies to build stylistic profiles with very good results (35, 69). SuperStyl use internally the SVM and other pipeline facilities from scikit-learn (74).

**Figure 1:**
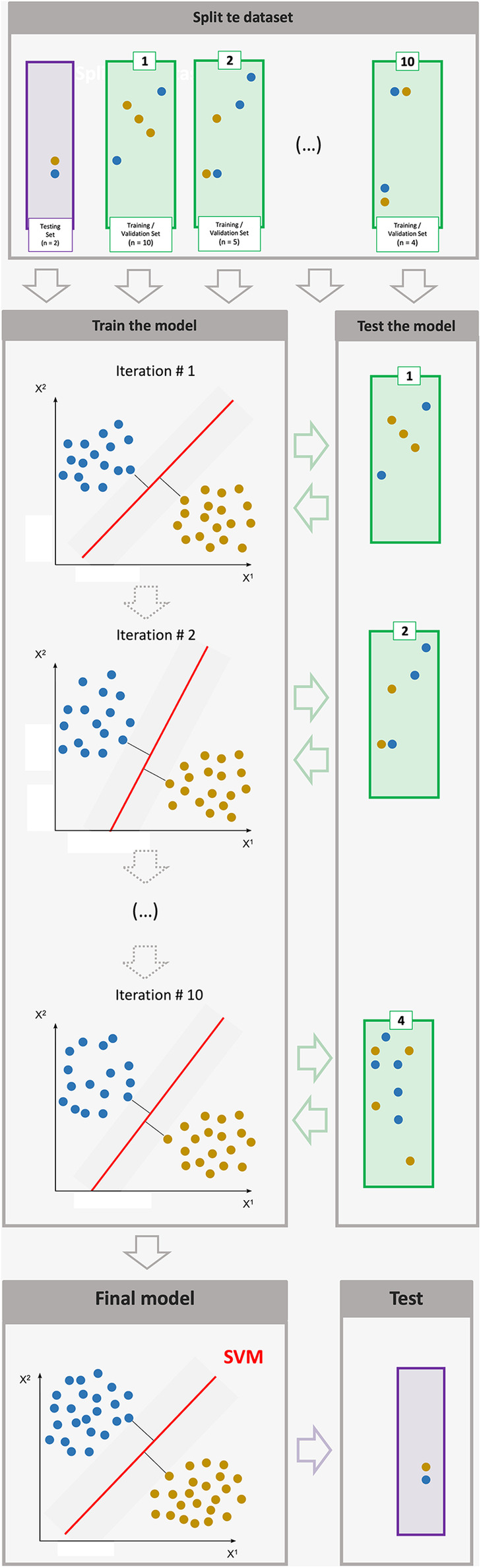
Each of the sub-corpora is divided into k samples (here 10) of similar length, so as to iteratively train the algorithm on all samples minus one, this last sample allowing each iteration to evaluate the result obtained with the k-1 others (here 9). During training, the machine statistically determines a separation boundary (a “hyperplane”) between several classes (here the ADHD and control groups). In order to check whether the model produced is viable, we use a test set, which was not seen during training.

Training a SVM (cf. Figure 1) involves three important steps: (1) selecting features, (2) tuning hyperparameters, and (3) understanding evaluation metrics (75).

#### Features

We test two features:

(1) Function words (FW) are words that carry minimal semantic meaning but play a grammatical role in the sentence, including articles, prepositions, conjunctions, and auxiliary verbs (40). They are extremely valuable because (i) their large number makes them particularly useful for statistical analyses, (ii) their use is done in a more unconscious way, (iii) they are not related to the content and, for instance, allow comparisons between texts with different themes (76). Function words have been found to correlate with gender, age, emotional states and reactions to stressors (40, 77, 78).
(2) Characters 3-grams are a sequence of three consecutive characters within a string of characters (79, 80). Each character 3-grams represents a sliding window of three characters moving through the adolescent’s narrative. For example, if the latter said “During the night (…),” the character 3-grams will be dur, uri, rin, ing, ng_ and so on. Character 3-grams capture subword level patterns, providing a granular view of textual features and capturing subtle stylistic nuances despite the short size of a given text (76, 81).

#### Hyperparameters

Because the length of SDMs varies a lot and we have more SDMs in the control group than in the ADHD groups, we test different resampling methods [undersampling the minority class, oversampling the majority class; cf. (82)] and the use of class weights. Rather than using the entire corpus, it is divided into samples of *n* words, and several sizes are tested (1’000, 1’250, 1’500 and 2’000). All tests are conducted with a linear kernel and a 10-fold validation, on data normalized applying Euclidean vector length normalization (L2 normalization) using z-scores for variables (33).

#### Evaluation metrics

Key metrics used to evaluate the performance of an SVM model are:

**Precision** (also known as Positive Predictive Value) is calculated as the proportion of positive predictions that were actually correct (83):

(5) $$\mathrm{Precision}=\frac{\mathrm{True}\;\mathrm{Positives}}{\mathrm{True}\;\mathrm{Positives}+\mathrm{False}\;\mathrm{Positives}}$$

**Recall** (also known as Sensitivity or True Positive Rate) is calculated as the proportion of true positive predictions among all actual positive instances (83):

(6) $$\mathrm{Recall}\;=\;\frac{\mathrm{True}\;\mathrm{Positives}}{\mathrm{True}\;\mathrm{Positives}\;+\;\mathrm{False}\;\mathrm{Negatives}}$$

**The F1 score** is the most common measure used on imbalanced classification tasks (84). It provides a single metric balancing both precision and recall. It is calculated using the following formula:

(7) $$F1\;\mathrm{Score}=2\times \frac{\mathrm{Precision}\times \mathrm{Recall}}{\mathrm{Precision}+\mathrm{Recall}}$$

**Accuracy** is computed as the proportion of correct predictions (true positives + true negatives) made by the model out of all predictions (true positives + true negatives + false positives + false negatives) (83):

(8) $$\mathrm{Accuracy}=\frac{\text{Number of correct predictions}}{\text{Total number of predictions}}$$

After running the two models (one with FW and the other with characters 3-grams), the results were examined by investigating the lexical information of the corpus and unfolding the SVM to identify its key features (i.e., the text features used by the model to make the classification).

## Results

As outlined in the Methods section, we computed four linguistic metrics. The corresponding results are presented below and summarized in Table 2.

**Table 2:** Mean and standard deviation (SD) of linguistic measures for ADHD and control groups.

Measure	Group	Mean	SD
Wordcount	ADHD	71.667	32.493
	Control	104.069	41.279
Lexical diversity (ALD)	ADHD	0.727	0.074
	Control	0.746	0.051
Lexical density (MATTR)	ADHD	0.805	0.023
	Control	0.811	0.023
Cohesion score (CS)	ADHD	66.7	29.2
	Control	97.0	30.4

### Wordcount

As defined in the *Methods*, the total wordcount was computed for each sample (see Equation 1). A Wilcoxon rank-sum test, comparing the wordcount between the ADHD group and the control group, indicated a significant difference (*p*-value < 0.005), with the ADHD group generating significantly shorter texts than the control group.

### Lexical diversity

A Wilcoxon rank-sum test was performed to compare the distributions of Average Lexical Diversity (ALD) between the ADHD group and the control group. The results of the test indicated that the observed difference in the mean between the two groups was statistically significant (*p*-value < 0.0001). This indicated that the ALD values for the ADHD group were significantly lower than those of the control group.

### Lexical density

A Wilcoxon rank-sum test was performed to compare the distributions of the Moving-Average Type-Token Ratio (MATTR) between the ADHD group and the control group. The results of the test indicated that the observed difference in the mean between the two groups was statistically significant (*p* < 0.0001). This result indicated that the MATTR values for the ADHD group were significantly lower than those of the control group.

### Cohesion

A Wilcoxon rank-sum test was performed to assess the statistical significance of the difference in Cohesion Score (CS) between the ADHD and control groups. The test results demonstrated that the difference in mean CS between the two groups was highly statistically significant (*p*-value < 0.0001), with the ADHD group exhibiting lower CS compared to the control group.

### Support vector machine (SVM)

The performance of the SVM model was evaluated using four key metrics (see Methods section): Precision, Recall, F1 Score, and Accuracy. The computational procedures for these metrics are detailed in Equations 5–8, respectively, and summarized in Table 3. Based on these evaluation criteria, the best results were achieved with 1,500- word samples, class weighting, and Tomek Links for Function Words (FW), and with 1,750-word samples, class weighting, and downsampling for 3-grams. Accuracy was lower with FW (85%) than with 3-grams (100%), the latter emerging as a particularly promising indicator for research in psycholinguistics. However, the results remained surprisingly good in both cases: the SVM effectively recognized the ADHD and the control groups, highlighting a distinct linguistic signal in SDMs of adolescents with ADHD. The recall for the ADHD group warranted particular attention. In a screening context, maximizing the true positive rate is essential to avoid missing individuals with ADHD. In fact, in this scenario, false positives are acceptable, as they can be excluded through further clinical assessment, whereas undetected cases are problematice, as they may go untreated. From this perspective, the 75% recall for FW was suboptimal, as it meant that approximately one-quarter of ADHD cases were missed.

**Table 3:** SVM results for function words (FW) and character 3-grams.

Class	Precision	Recall	F1-score	Support
Function words (FW)				
Control	0.89	0.89	0.89	9
ADHD	0.75	0.75	0.75	4
Accuracy			0.85	13
Character 3-grams				
Control	1.00	1.00	1.00	7
ADHD	1.00	1.00	1.00	3
Accuracy			1.00	10

#### Main markers of both groups

**Figure 2:**
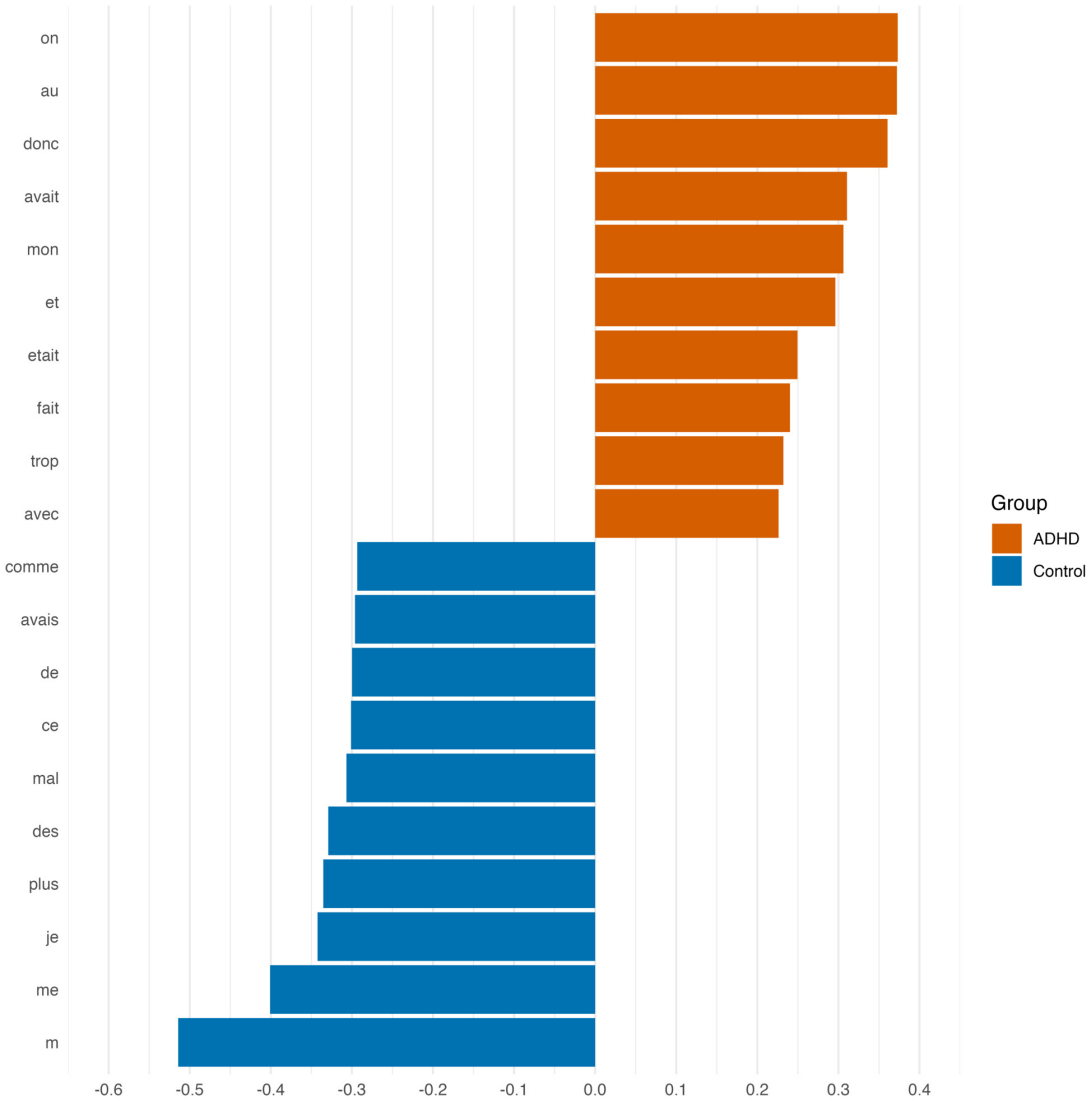
Most important FW for stylometric classification.

For the ADHD group, the main FW markers (see Figure 2) included the neutral pronoun (“on”) combined with third-person auxiliaries and an abundance of words with syntactic function (“donc,” “et,” “avec”). On the other hand, the control group was found to be strongly marked by the first-person pronoun (“je,” “me/m”), the adverb of quantity “plus,” and the indefinite article “des.”

**Figure 3:**
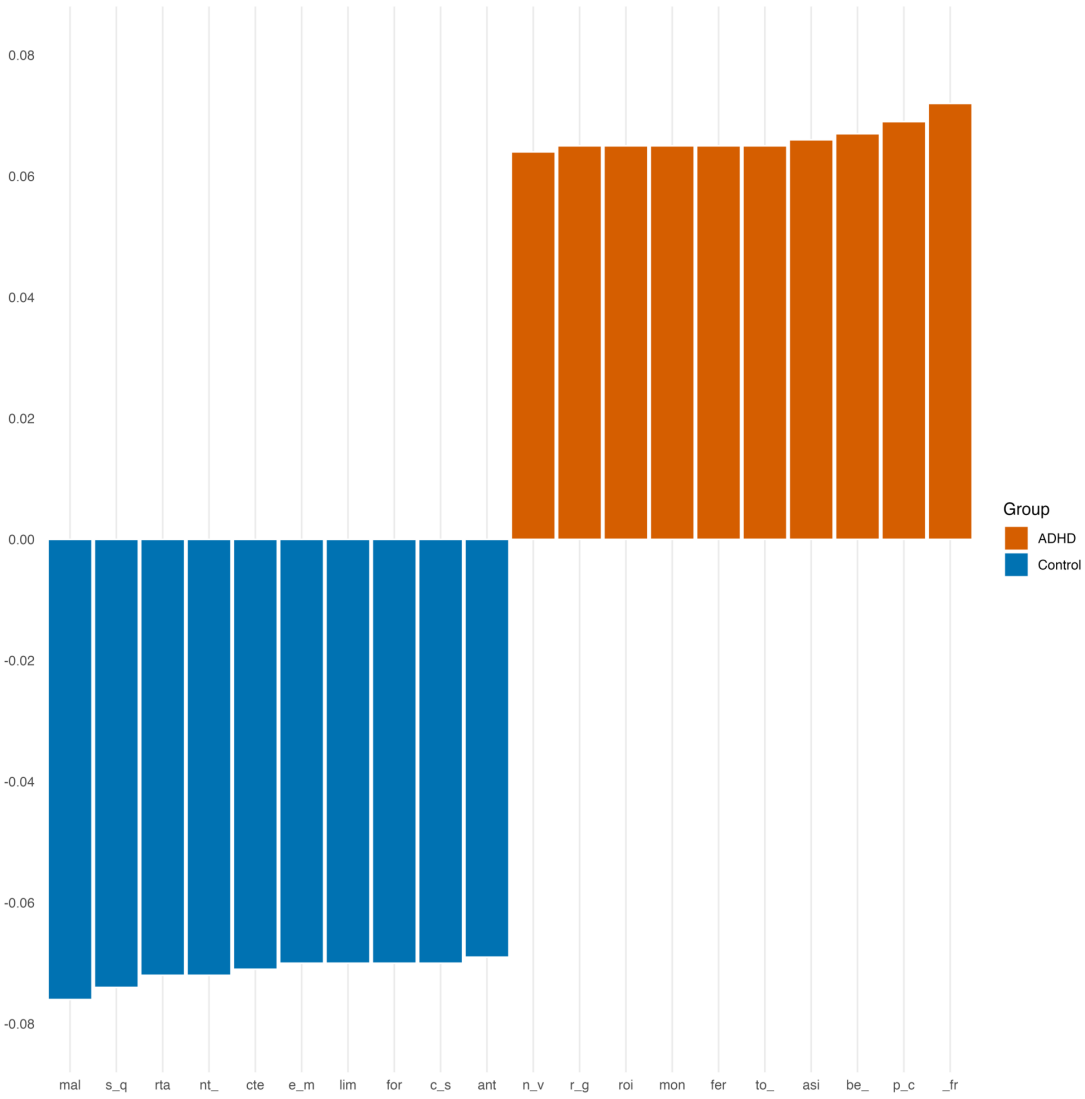
Most important 3-grams for stylometric classification.

As for 3-grams, the main markers for both groups are shown in Figure 3.

## Discussion

This study targeted three main objectives. First, to use NLP to characterize the linguistic features of SDMs (i.e., wordcount, lexical diversity, lexical density and cohesion) in adolescents with ADHD compared to a control group. Second, to quantify the predictive power of stylometry for classifying ADHD vs. control individuals. And third, to qualitatively analyze key linguistic markers, to further the scientific basis of automated text-based screening for ADHD.

For Wordcount, the ADHD group produced significantly shorter text narratives compared to the control group. This result could be explained by difficulties observed in individuals with ADHD related to their pragmatic language skills (85), and could be related with their difficulties in communicating effectively in social settings (86). However, further analysis are required to understand the relationship between wordcount and pragmatic language skills.

We also found significant differences in Lexical Diversity and Lexical Density where, in both cases, the ADHD group shows lower mean scores. Lower lexical diversity can affect communication skills and have a negative impact in social interactions and academic performance, both of which are associated with ADHD (87).

Lower lexical density in individuals with ADHD may be indicative of their cognitive and attentional profile, often characterized by difficulties in sustaining attention and deficits in executive function, which can lead to challenges in encoding, retrieving, and organizing information. These cognitive limitations can adversely affect communication by reducing language production and diversity, potentially impairing overall communication abilities and limiting social interactions and academic performance. Although these findings suggest that lexical density is a useful metric for evaluating communication skills in populations with developmental or cognitive challenges, further analysis is required to fully understand the practical implications and underlying causes of these differences between groups.

These findings in both lexical diversity and lexical density underscore the importance of intervention programs aimed at improving language skills and self-knowledge in adolescents with ADHD. Indeed, targeted interventions in these two topics may significantly boost communication skills and foster better self-regulation, which in turn could positively impact social interactions and academic performance (86, 88, 89). However, an important point to underline here is that the focus of attention should not be on vocabulary per se, but in finding more words about the topic of the task of the SDM, i.e., personal and significant events related with narrative identity. This involves exploring which thoughts and feelings had emerged at that moment and could be done in a simple way by modifying the task of the SDMs with a semi-structured questionnaire oriented to explore and refine the contents of individual narratives.

Participants in the ADHD group also exhibit significantly lower scores in cohesion measures, indicating less structured language, which is also associated with difficulties in executive functioning (90) and working memory impairments (91). Indeed, the latter may hinder the ability to retain and manipulate information over short periods, directly impacting the coherence of verbal and written communication. Moreover, sustained attention deficits exacerbate this issue, as individuals with ADHD may lose track of the narrative, resulting in fragmented and less cohesive discourse. These findings highlight the need for targeted interventions addressing these cognitive deficits. Indeed, enhancing executive functioning through cognitive training and therapeutic strategies could improve language cohesion and overall communication skills in individuals with ADHD.

Regarding the capacity of stylometry to detect the SDMs of adolescents with ADHD at the group level, we found that the SVM classifier successfully differentiated between the autobiographical texts of adolescents with ADHD and those of the control group. The results can be considered robust, given that our lowest accuracy score was 85% using function words, while the highest accuracy score reached 100% with characters 3-grams. Of note, and for diagnostic purposes, a model with an accuracy exceeding 80% is considered to have very good performance (92). Consequently, these findings indicate that stylometric analysis is a viable method for detecting group-level differences in narrative writing between adolescents with ADHD and their non-ADHD peers. Indeed, our study confirms that adolescents with an ADHD diagnosis do have a distinct narrative style, as evidenced by significant differences in the language patterns of their autobiographical narratives compared to the control group.

Last but not least, distinct markers for each group were identified, which hold significant psychological meaning. With respect to personal pronouns used in both groups, one of the main markers for the ADHD group was the indefinite neuter pronoun “on.” This result is particularly intriguing given that the SDMs task requires individuals to recall personally significant memories, which are naturally expected to evoke first-person (“I” or “me”) experiences. In French, the personal pronoun “on” refers to the narrator and one or more other persons without having explicitly or necessarily mentioned them (93). Translating “on” into English typically requires choosing between “one” or “we,” depending on the context [cf. (94)]. Although “one” is commonly used (Ibid.), the adolescents in this study frequently describe situations involving their peer groups in their SDMs. Consequently, “we” better captures the collective identity and shared experiences implied, making it the more appropriate translation in this case. In the control group the first singular pronoun “je” (“I”) was the main marker. According to Boulard and Gauthier (95), the emergence of this personal pronoun in children’s narratives enables first-person storytelling and is essential for developing self-awareness and differentiation through language. Once children move beyond the individuation stage during adolescence, individuals go through significant psychological changes as they form their identity and establish a sense of self that is distinct from their parents and peers (96). At that developmental stage, the use of personal pronouns provides information about the narrator’s focus and self-perception in relation to their social environment [cf. (97)]. According to Sutin and Robins (98), in the first-person perspective, individuals see the event through their own eyes, while a reduced use of the first person may serve a distancing function helping to reduce emotional reliving and to distance the current self from the self in the recalled event. Consequently, and according to this model, the marker “on” could reveal difficulties in experiencing affects and/or a need to distance oneself from the latter reflecting difficulties in emotion regulation. Several studies have found evidence of the impact of emotional dysregulation in youth with ADHD. In fact, these children and adolescent are six times more likely to have emotional regulation difficulties than their non-affected siblings (99). These findings underscore the importance of enhancing self-focus in interventions aimed at improving self-awareness among adolescents with ADHD, thereby enhancing their ability to embody the present, which means to be focused and attentive in the “here and know” from a mind body connection perspective as is the case in mindfulness based therapy.

In conclusion, the results of this study supports the hypothesis that ADHD impacts narrative construction in measurable ways, potentially reflecting broader cognitive and linguistic differences. The use of computational linguistics, including NLP and stylometry, demonstrates the utility of machine learning approaches in psychological research, particularly for identifying subtle linguistic markers associated with mental health disorders. By identifying patterns in language use, clinicians can gain insights into the cognitive and linguistic profiles of individuals with ADHD, aiding in diagnosis and the tailoring of interventions helping to provide more targeted and effective therapeutic strategies. In this sense, these findings have implications for developing new diagnostic tools and enhancing our understanding of the cognitive and linguistic profiles of individuals with ADHD.

While the use of stylometric methods for ADHD screening and diagnosis shows promise, several methodological improvements are needed to enhance their precision and automation. Despite these limitations, our findings highlight the potential of stylometry in both narrative identity research and in ADHD diagnostics. Stylometric analysis may offer a valuable tool for detecting subtle linguistic markers associated with mental health conditions. As these methods continue to evolve, their ability to classify narratives at both the group and individual level opens new possibilities for innovative forms of psychological assessment. However, further research is required to identify the most informative features specific to ADHD and to validate these findings in larger, more diverse samples that contain other neurodevelopmental conditions such as specific learning disorder and autism.

This study has several limitations that should be addressed in future research. Firstly, the sample size is small, which may affect the generalizability of the findings. Secondly, while 16% of participants in the ADHD group had a diagnosed learning disorder, the exclusion criteria required the absence of any clinical diagnoses for the control participants. Future studies with larger groups should assess the impact of learning disorders on the present results. Third, the methodology is limited to SDMs, while no other types of narratives have been tested. This restricts the scope of our analysis and may overlook other relevant linguistic features. Additionally, the study did not include analyses examining the influence of ADHD severity on classification performance, nor whether the identified linguistic markers were associated with general verbal abilities, ADHD subtypes, or social communication skills. This limitation is primarily due to the sample size, which may have constrained our ability to capture meaningful variation within the ADHD population. As a result, the current findings cannot definitively isolate linguistic features that are specific to ADHD, as the dataset may reflect the influence of comorbidities commonly associated with the condition.

While we cannot quantify the individual contribution of these comorbid factors to the classification outcomes, it is important to emphasize that ADHD is, by definition, a heterogeneous disorder that frequently co-occurs with other neurodevelopmental and mental health conditions. From a screening perspective, this heterogeneity does not diminish the value of our approach; rather, it underscores the potential of stylometric analysis as a broad initial filter. The ability to detect linguistic patterns characteristic of individuals within the ADHD spectrum—regardless of comorbid presentations—may serve as a valuable first step in identifying at-risk individuals who warrant further clinical assessment.

Addressing these issues is crucial to enhance the validity and utility of computational linguistic analysis in diagnosing and understanding ADHD.

Future research involving larger and more diverse samples—including comparative analyses with other clinical populations such as individuals with autism spectrum disorder or learning disabilities—will be essential to determine the specificity and diagnostic utility of these linguistic markers. Such work will be crucial for advancing this tool beyond initial screening, refining it toward diagnostic applications that align with both categorical and dimensional perspectives on ADHD.

To increase the accuracy and fine-grained detection of ADHD, several avenues for future research should be explored. First, investigating potential language marker differences based on gender is imperative. Additionally, refining detection precision to identify distinct modalities of ADHD, such as hyperactive modalities, is crucial. This entails incorporating a larger sample size, in different languages (specifically English), including more hyperactive participants, to capture broader and more representative characteristics accompanying ADHD.

## Data Availability

The raw data supporting the conclusions of this article will be made available by the authors, without undue reservation.

## Footnotes

^1^Thorne A, McLean KC. Manual for Coding Events in Self-De!ning Memories. Santa Cruz: University of California (2001).
